# Two Pentatricopeptide Repeat Proteins Are Required for the Splicing of *nad5* Introns in Maize

**DOI:** 10.3389/fpls.2020.00732

**Published:** 2020-06-03

**Authors:** Huanhuan Yang, Zhihui Xiu, Le Wang, Shi-Kai Cao, Xiulan Li, Feng Sun, Bao-Cai Tan

**Affiliations:** Key Laboratory of Plant Development and Environmental Adaptation Biology, Ministry of Education, School of Life Sciences, Shandong University, Qingdao, China

**Keywords:** PPR proteins, group II intron splicing, *nad5*, complex I biogenesis, mitochondria, maize seed development

## Abstract

Mitochondrial genes in flowering plants contain predominantly group II introns that require precise splicing before translation into functional proteins. Splicing of these introns is facilitated by various nucleus-encoded splicing factors. Due to lethality of mutants, functions of many splicing factors have not been revealed. Here, we report the function of two P-type PPR proteins PPR101 and PPR231, and their role in maize seed development. PPR101 and PPR231 are targeted to mitochondria. Null mutation of *PPR101* and *PPR231* arrests embryo and endosperm development, generating *empty pericarp* and *small kernel* phenotype, respectively, in maize. Loss-of-function in PPR101 abolishes the splicing of *nad5* intron 2, and reduces the splicing of *nad5* intron 1. Loss-of-function in PPR231 reduces the splicing of *nad5* introns 1, 2, 3 and *nad2* intron 3. The absence of Nad5 protein eliminates assembly of complex I, and activates the expression of alternative oxidase AOX2. These results indicate that both PPR101 and PPR231 are required for mitochondrial *nad5* introns 1 and 2 splicing, while PPR231 is also required for *nad5* intron 3 and *nad2* intron 3. Both genes are essential to complex I assembly, mitochondrial function, and maize seed development. This work reveals that the splicing of a single intron involves multiple PPRs.

## Introduction

Mitochondria are highly metabolically active organelles in eukaryotic cells that perform multiple cellular functions, including basic energy supply and redox regulation, ion transmembrane transport, and metabolic pathways integration (Sweetlove et al., 2007). Mitochondria are derived from ancient α-proteobacteria via endosymbiosis and the majority of bacterial genes are either transferred to the host nuclear genome or lost during evolution (Martin and Herrmann, 1998). The present mitochondria in higher plants host only 5% of the genes encoding proteins essential for mitochondrial biogenesis and functions (Gray et al., 1999; Martin et al., 2012). For example, the maize mitochondrial genome contains 58 genes encoding 22 proteins for respiratory chain, 9 ribosomal proteins (RP), a transporter (MttB), a maturase (Mat-r), 3 rRNAs (rrn5, rrn18, rrn26), and 21 tRNAs (for 14 amino acids) (Clifton et al., 2004). Moreover, expression of the mitochondrial genes is strictly regulated by the nuclear genome, particularly at post-transcriptional level which includes intron splicing, RNA editing, RNA cleavage, RNA stabilization and translation (Fujii and Small, 2011; Barkan and Small, 2014).

Except for *cox1* that contains a group I intron in some angiosperm species, most of the mitochondrial genes in flowering plants only contain group II introns (Brown et al., 2014). Most of them are *cis*-spliced introns that the splicing occurs in one mRNA molecule. Some group II introns, however, are fragmented and transcribed as separate RNAs, which are spliced in *trans* configuration (Bonen, 2008; Brown et al., 2014). Group II introns typically consist of six stem-loops, DI-DVI, of which DI, DV, and DVI are crucial to intron splicing (Novikova and Belfort, 2017). Typical group II introns are also mobile genetic elements which can reversely transcribe and insert back into the host genome, referred as “retrohoming” (Eickbush, 1999). Bacterial group II introns can self-splice under high-salt concentrations *in vitro*, but *in vivo* the splicing is facilitated by the cognate intron-encoded maturase (Mat) (Pyle, 2016). Higher plant organellar introns, however, have lost the self-splicing capability due to mutations in intron sequences, rearrangement, and loss of most maturase genes during evolution (Schmitz-Linneweber et al., 2015). In addition, most intron specific maturase genes have been lost, with only a *matK* gene retained in the *trnK* intron in plastids and a *matR* gene retained in the 4th intron of *nad1* in mitochondria (Clifton et al., 2004; Brown et al., 2014). For these reasons, intron splicing in higher plant organelles requires a large number of nuclear-encoded RNA-binding factors in addition to the maturases. Recent studies have indicated that multiple families of RNA binding proteins are involved in intron splicing. In plant organelles, these include plant organellar RNA recognition (PORR) protein (Kroeger et al., 2009; Francs-Small et al., 2012), DEAD-box RNA helicase (Köhler et al., 2010), regulator of chromosome condensation-like (RCC) protein (Kühn et al., 2011), RAD-52-like protein (Samach et al., 2011), chloroplast RNA splicing and ribosome maturation (CRM) proteins (Zmudjak et al., 2013), mitochondrial transcription termination factor (mTERF) proteins (Hammani and Barkan, 2014) and pentatricopeptide repeat (PPR) proteins (Barkan and Small, 2014).

PPRs are a large family of nuclear-encoded proteins widespread in land plants, with 400 to 600 *PPR* genes in most angiosperm genomes (Lurin et al., 2004; Cheng et al., 2016). PPRs consist of 2 to 30 tandem repeats of a degenerate 35-amino-acid motif that forms an anti-parallel α- helix (Yin et al., 2013). Based on their constituent motifs, PPRs are divided into P-type and PLS-type subfamilies. The P-type subfamily contains only P motifs, and PLS-type subfamily contains long (L, 35 or 36 amino acids) and short (S, 31 amino acids) motifs. Based on the C terminal domain, PLS-type subfamily is further classified into E, E+, and DYW subgroups (Claire et al., 2004). PLS-type PPR proteins are predominantly involved in RNA editing (Liu et al., 2013; Li et al., 2014, 2019; Sun et al., 2015; Yang et al., 2017), whereas P-type PPR proteins participate in intron splicing (Liu et al., 2010; Hsieh et al., 2015; Xiu et al., 2016; Ren et al., 2017; Sun et al., 2018; Yang et al., 2019), RNA stability (Haili et al., 2013; Lee et al., 2017; Wang et al., 2017; Zhang et al., 2017), and translation (Cohen et al., 2014; Haili et al., 2016).

In plants, most of mitochondrial introns reside in *nad* (NADH dehydrogenase) genes, which encode subunits of complex I in mitochondrial respiratory chain (Li-Pook-Than and Bonen, 2006). Maize mitochondrial genome contains 22 group II introns, of which 19 reside in *nad* genes, *nad1*, *nad2*, *nad4*, *nad5* and *nad7*, and 3 in *rps3*, *cox2*, and *ccmFc* genes. And five of them are *trans* introns (Burger et al., 2003; Clifton et al., 2004). Previous studies have reported the P-type PPRs participating in intron splicing of mitochondrial *nad* genes. In Arabidopsis, OTP43 is required for the splicing of *nad1* intron 1 and *nad2* intron 1 (De Longevialle et al., 2007), ABO5 and MISF26 for *nad2* intron 3 (Liu et al., 2010; Wang et al., 2018), OTP439 for *nad5* intron 2 and TANG2 for *nad5* intron 3 (Francs-Small et al., 2012), BIR6 for *nad7* intron1 and SLO3 for *nad7* intron 2 (Koprivova et al., 2010; Hsieh et al., 2015). In maize, EMP11 and DEK2 are involved in *nad1* introns splicing (Qi et al., 2017; Ren et al., 2017). EMP16, EMP10, DEK37 and EMP12 are required for *nad2* introns splicing (Xiu et al., 2016; Cai et al., 2017; Dai et al., 2018; Sun et al., 2019). DEK35, DEK41, DEK43, and EMP602 are implicated in *nad4* introns splicing (Chen et al., 2017; Ren et al., 2019, 2020; Zhu et al., 2019). The loss-of-function in these PPRs gives rise to splicing deficiency, and impedes the assembly and activity of complex I, thus disrupting the function of mitochondrial respiratory chain. These results imply the essential effects of PPRs on intron splicing and mitochondrial metabolism. However, the unknown molecular function of a large number of PPRs and the mechanism of intron splicing still need more research.

In this study, we reveal the function of two P-type PPR proteins on the splicing of multiple *nad5* introns through a genetic and molecular characterization of *ppr101* and *ppr231* mutants. Interestingly, both PPR101 and PPR231 are required for the splicing of *nad5* introns 1 and 2, suggesting that splicing of a single intron requires more than one PPR proteins. These two proteins may not interact physically. We further prove that PPR101 and PPR231 are essential to mitochondrial complex I biogenesis and seed development in maize. This work adds to body of information on PPR functions and provides clue to the elucidation of intron splicing in mitochondria.

## Materials and Methods

### Plant Materials

The two alleles of the *ppr101* and *ppr231* mutants were isolated from the UniformMu stocks (Mccarty et al., 2013) and backcrossed to the W22 inbred genetic background. The mutator insertion was confirmed by PCR amplification using gene-specific primers and *Mu*-specific primers. The mutants were kept in heterozygotes condition and maize plants were grown in the experimental field in Qingdao, Shandong Province and Huangliu, Hainan Province under natural growth conditions.

### DNA Extraction and Linkage Analysis

Genomic DNA of young maize leaf was extracted by urea-phenol-chloroform-based extraction method as described previously (Tan et al., 2011). For linkage analysis experiment, the leaf genomic DNA was extracted from individual plant and used as templates for PCR analysis. Each plant was self-pollinated and the progeny was observed for segregation of mutant kernels at 25% ratio.

### Light Microscopy of Cytological Sections

Wildtype and mutant kernels were obtained from heterozygous maize plants which were self-pollinated at 9 DAP and 15 DAP. The fixed kernels were sectioned after dehydrated, infiltrated, embedded, de-paraffinized and stained as described previously (Shen et al., 2013).

### Subcellular Localization of PPR101 and PPR231

The first 400-bp fragment of *PPR101* and 615-bp of *PPR231* in coding region without the stop codon were amplified from maize cDNA of the W22 inbred line. PCR products were cloned into pENTR/D-TOPO vector (Invitrogen), and then integrated into binary vector pGWB5 by LR site-specific recombination. The destination plasmid pGWB5-PPR101**^N200^**-green fluorescent protein (GFP) (pGWB5-PPR101**^N200^**-GFP) and pGWB5-PPR231**^N205^**-green fluorescent protein (GFP) (pGWB5-PPR231**^N205^**-GFP) were then introduced into *Agrobacterium tumefaciens* strain EHA105 and infiltrated into *Nicotiana tabacum* leaf epidermal cells as described (Herpen et al., 2010). The co-localization of GFP signal and MitoTracker Red were observed after 24–36 h with an Olympus FluoView FV1000 confocal microscope. MitoTracker Red (Thermofisher Scientific)^1^ was used as the mitochondrion maker.

### RNA Extraction, RT-PCR and Quantitative RT-PCR

Total RNA was extracted by using the RNeasy Plant Mini Kit (QIANGE)^2^ from 100 mg of kernels samples according to the protocol. RNA was further treated with RNase-free DNase I (ThermoFisher Scientific, Carlsbad, CA, United States) to remove residual DNA contamination. The reverse transcription PCR was performed according to the TransScript First-Strand cDNA Synthesis SuperMix (TRANSGEN BIOTECH) instructions. Expression level of *PPR101* and *PPR231* in the WT and different alleles was analyzed by primers PPR101-F1/R1 and PPR231-F1/R1. *ZmActin* (GRMZM2G126010) was used as normalization. Expression pattern of *PPR101* and *PPR231* in various maize tissues was analyzed by qRT-PCR using primers PPR101-RT-F1/R1 and PPR231-RT-F1/R1. Primers are listed in Supplementary Table S1.

Quantitative real-time polymerase chain reaction (qRT-PCR) assay was performed with SYBR Green according to the manufacturer’s instruction (Bio-Rad)^3^. cDNA was synthesized using 500 ng total RNA in a 25 μl reaction and then diluted 10 times. After normalization against *ZmActin* to account for RT efficiency differences, roughly 2 ng RNA (derived cDNA) was used as a template in a 15 μl reaction for qRT-PCR amplification in a Bio-Rad CFX96 Touch Light Cycler. The relative quantification of gene expression was calculated by the 2^–ΔΔCt^ method. *ZmActin* (GRMZM2G126010) reference gene was used to normalize the expression. For each qRT-PCR experiment, three biological replicates and three technical replicates were analyzed. The primers were used as described (Li et al., 2014). Additional primers are listed in Supplementary Table S1.

### Functional Analysis of *PPR101* and *PPR231*

Total RNA was extracted from embryos and endosperms of the wildtype, *ppr101* and *ppr231* mutant kernels at 12 DAP and gDNA removal was carried out as described above. RNA without gDNA contamination was reverse transcribed with random hexamers. The expression level of 35 maize mitochondrial transcripts in the WT, *ppr101* and *ppr231* mutants was analyzed by RT-PCR with primers as described previously (Liu et al., 2013). The splicing efficiency of 22 mitochondrial group II introns between the WT, *ppr101* and *ppr231* mutants was compared by RT-PCR and qRT-PCR. *ZmActin* was used as normalization. Primers are listed in Supplementary Table S1.

### BN-PAGE, Mitochondrial Complex I Activity and Western Blotting

Crude mitochondria of the embryo and endosperm from 9 to 14 DAP of the wildtype and mutant kernels were extracted as described (Li et al., 2014). Blue native-polyacrylamide gel electrophoresis (BN-PAGE) and in-gel complex I activity assay were carried out as described previously (Sun et al., 2015). Protein abundance was detected by western blotting assay with antisera, including Nad9, Cox2, Cyt*c*1, ATPase α-subunit and AOX as described previously (Sun et al., 2015; Xiu et al., 2016).

### Yeast Two-Hybrid Assay

The yeast two-hybrid (Y2H) assays were performed as described previously. DNA fragments without signal peptides of PPR101 and PPR231 were recombined into plasmids pGADT7 and pGBKT7. Plasmids were co-transformed and sprayed on dropout medium of DDO (SD/-Leu/-Trp), TDO (SD/-Leu/-Trp/-His), and QDO (SD/-Leu/-Trp/-His/-Ade).

## Results

### PPR101 and PPR231 Are P-Type PPR Proteins Targeted to Mitochondria

**FIGURE 1:**
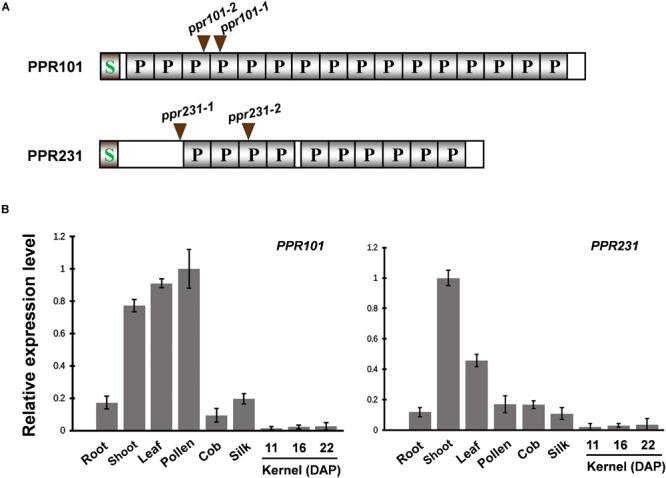
Expression of *PPR101* and *PPR231*. **(A)** Schematic presentation of PPR101 and PPR231. Two *Mutator* (*Mu*) insertion sites are labeled by brown triangles. P motifs are predicted by TPRpred (https://toolkit.tuebingen.mpg.de/#/tools/tprpred). S, signal peptide. **(B)** Quantitative RT-PCR analysis of *PPR101* and *PPR231* expression in major maize tissues and developing kernels. *ZmActin* was used to normalize the quantifications. Values and error bars were means of three technical replicates, ±SD. DAP, days after pollination.

To characterize the function and the role of PPR proteins in plant growth and development, we analyzed multiple *PPR* genes and identified mutants from the UniformMu mutagenesis population (Mccarty et al., 2013). Two PPR genes are focused as they all encode P-type PPR proteins and are predicted to localize in mitochondria. PPR101 (GRMZM2G023999) contains 647 amino acids with 16 PPR motifs, and PPR231 (GRMZM2G018757) 526 amino acids with 10 PPR motifs (Figure 1A). A signal peptide was found in the N-terminus of each protein and no other domains were identified.

To examine the expression pattern of *PPR101* and *PPR231*, quantitative RT-PCR (qRT-PCR) analyses were performed on major maize tissues and developing kernels. Results indicate that these two genes are expressed in various tissues with slightly different expression patterns (Figure 1B). The expression of *PPR101* is relatively high in pollen, leaf and shoot, and low in root, silk, cob, especially in developing kernels. The expression of *PPR231* is high in shoot, with relatively low in other tissues and developing kernels. These data suggest that rather than seed specific, these two PPRs are constitutive genes that have functions throughout plant growth and development. However, due to the embryo lethality caused by these two genes mutation, the influences in other tissues cannot be examined.

**FIGURE 2:**
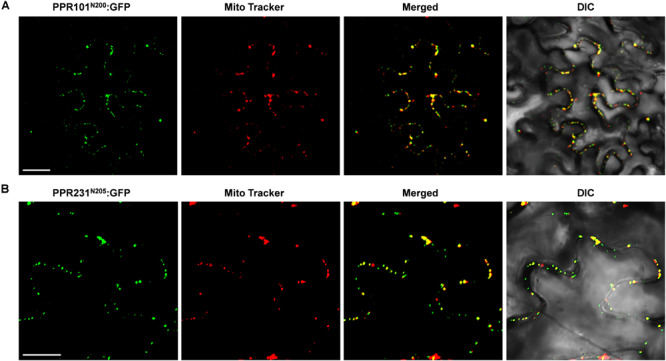
Subcellular localization of PPR101 and PPR231. PPR101 and PPR231 are localized in mitochondria. The truncated protein with 200 amino acids of PPR101 **(A)** and 205 amino acids of PPR231 **(B)** were fused with GFP and transiently expressed in tobacco leaf epidermal cells and the GFP signals were detected by confocal fluorescent microscopy. Mitochondria are marked with MitoTracker. DIC, differential interference contrast; GFP, green fluorescence protein; N^200^, the N-terminal 200 amino acids of PPR101; N^205^, the N-terminal 205 amino acids of PPR231. Scale bar = 20 μm.

To experimentally determine the subcellular localization, we fused the full length PPR with GFP and transiently expressed the fusion protein in tobacco epidermal cells. However, expression of PPR101-GFP and PPR231-GFP did not produce any detectable signals. We then used the N-terminal 200 and 205 amino acids of PPR101 and PPR231, respectively, and fused with GFP. Expression of both constructs produced strong GFP signals in punctate dots that were merged with mitochondria as stained by MitoTracker Red, a marker of mitochondria (Figures 2A,B). These results suggest that PPR101 and PPR231 are targeted to mitochondria.

### Loss-of-Function Mutation in *PPR101* and *PPR231* Impairs Seed Development in Maize

**FIGURE 3:**
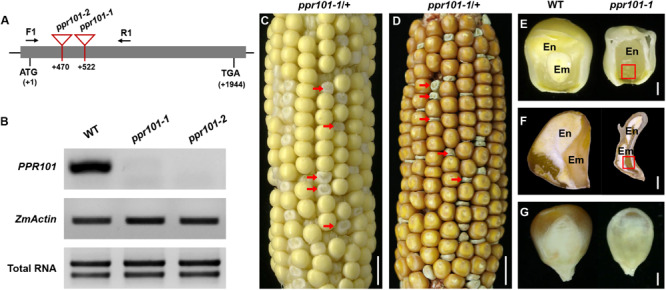
Mutant analysis of the *ppr101-1.* **(A)** Schematic presentation of the *PPR101* gene structure and positions of *Mutator* (*Mu*) insertions in two alleles. The gray box represents protein translated region, and black lines represent the 5′ and 3′ untranslated regions. *Mu* insertion sites of *ppr101* alleles are indicated by red triangles. **(B)** Semi-quantitative RT-PCR analysis of *PPR101* expression level in *ppr101* mutants and the wildtype siblings at 12 DAP with primers PPR101-F1/R1. Expression was normalized with total RNA and *ZmActin.* **(C,D)** The selfed ear segregates 3:1 for the WT and *ppr101-1* (red arrows) at 12 DAP **(C)** and mature stage **(D)**. Scale bar = 1 cm. **(E,F)** The view of endosperm and embryo of the WT and *ppr101-1* at 12 DAP **(E)** and mature stage **(F)**. En, endosperm; Em, embryo. Red box indicates the embryo. Scale bar = 1 mm. **(G)** Germinal side of the WT and *ppr101-1* mature kernels. Scale bar = 1 mm.

To reveal the function of these genes in plants, we isolated *Mutator* (*Mu*) insertional mutants from the maize UniformMu population (Mccarty et al., 2005). These seeds were planted and genotyped by PCR with gene specific primers and *Mu*-TIR primers. The insertion sites of each *Mu* element in the corresponding protein domains were indicated in Figure 1A. *PPR101* gene contains no intron and *PPR231* contains one intron. All the insertions are in coding region, thus disrupting the coding of proteins. For the *PPR101* gene, two alleles have the *Mu* insertions at 522 bp and 470 bp downstream from the ATG of *PPR101*, named *ppr101*-*1* and *ppr101-2*, respectively (Figure 3A). RT-PCR with primers across the insertion did not detect any expression of *PPR101* (Figure 3B), suggesting that wildtype transcripts cannot be produced in the mutants. Self-crossed heterozygous plants for *ppr101-1* and *ppr101-2* produced ears segregating *empty pericarp* (*emp*) and wildtype kernels in a ratio of 1:3 (102:314, *P* < 0.05). The *emp* phenotype is tightly linked to the *Mu* insertion as only the heterozygous plants produce *emp* kernels. Crosses between *ppr101-1* and *ppr101-2* heterozygotes also produce *emp* mutants, confirming that the *emp* phenotype is caused by the mutation of *PPR101* (GRMZM2G023999) (Supplementary Figure S1). Compared with the WT, the *ppr101-1* kernels are small during the entire seed development process and the pericarps are collapsed at maturity. Both the endosperm and embryo development are defective with its embryogenesis arrested at the transition stage and the endosperm development stalled in *ppr101-1*. As the maternal pericarp continues its growth, this causes a cavity between the pericarp and the zygote seed (Figures 3C–G).

**FIGURE 4:**
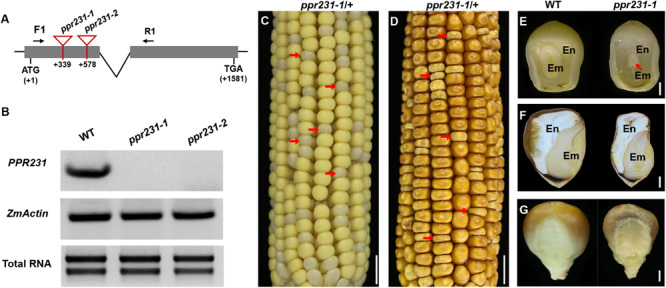
Mutant analysis of the *ppr231-1*. **(A)** Schematic presentation of the *PPR231* gene structure and positions of *Mutator* (*Mu*) insertions in two alleles. The gray boxes represent protein translated region, and black lines represent the 5′ and 3′ untranslated regions and intron. *Mu* insertion sites of *ppr231* alleles are indicated by red triangles. **(B)** Semi-quantitative RT-PCR analysis of *PPR231* expression level in *ppr231* mutants and the wildtype siblings at 12 DAP with primers PPR231-F1/R1. Expression was normalized with total RNA and *ZmActin.* **(C,D)** The selfed ear segregates 3:1 for the WT and *ppr231-1* (red arrows) at 12 DAP **(C)** and mature stage **(D)**. Scale bar = 1 cm. **(E,F)** The view of endosperm and embryo of the WT and *ppr231-1* at 12 DAP **(E)** and mature stage **(F)**. En, endosperm; Em, embryo. Red arrow indicates the embryo. Scale bar = 1 mm. **(G)** Germinal side of the WT and *ppr231-1* mature kernels. Scale bar = 1 mm.

For the *PPR231* gene, two alleles were isolated with *Mu* insertions at 339 bp and 578 bp downstream from the ATG, named *ppr231-1* and *ppr231-2* (Figure 4A). The selfed progeny of the *ppr231* heterozygous plants produced mutants at an approximately 25% ratio (137:406, *P* < 0.05). Molecular and genetic analysis indicate that the phenotype is caused by the mutation of *PPR231* (GRMZM2G018757) (Supplementary Figure S2). Wildtype *PPR231* transcripts were not detected in the mutants (Figure 4B). The *ppr231-1* mutant kernels show partially developed embryo and endosperm. No clear differentiation of shoot apex is detected in the embryo and the endosperm development is also delayed compared with the WT (Figures 4C–G). Both the *ppr101* and *ppr231* mutants are embryo-lethal and cannot germinate, implying that the two PPRs are essential to maize embryogenesis and endosperm development.

### Embryogenesis and Endosperm Development Are Arrested in *ppr101-1* and *ppr231-1*

**FIGURE 5:**
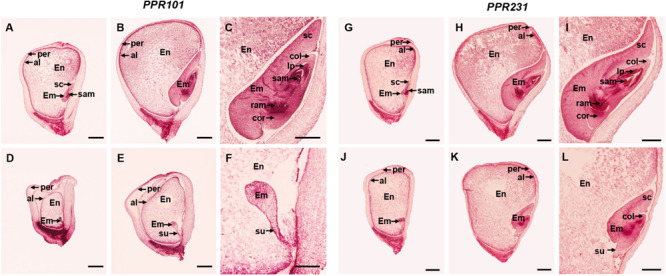
Comparison in kernel development between *ppr101-1*, *ppr231-1* mutant and wildtype sibling kernels. **(A–F)** Developmental analysis of the WT **(A–C)** and *ppr101-1* **(D–F)** at 9 DAP **(A,D)** and 15 DAP **(B,E)**. **(C,F)** are amplified embryo pictures of panels **(B,E)**. **(G–L)** Developmental analysis of the WT **(G–I)** and *ppr231-1* **(J–L)** at 9 DAP **(G,J)** and 15 DAP **(H,K)**. **(I,L)** are amplified embryo pictures of panels **(H,K)**. En, endosperm; Em, embryo; per, pericarp; al, aleurone; col, coleoptile; cor, coleorhizae; sam, shoot apical meristem; ram, root apical meristem; sc, scutellum; su, suspensor; lp, leaf primordia. Scale bars: **(A,B,D,E,G,H,J,K)** = 1.0 mm; **(C,F,I,L)** = 0.5 mm.

To further assess the developmental arrest in two mutants, we sectioned the homozygous and wildtype sibling kernels from the same segregating ear. At 9 DAP, the wildtype embryo differentiated a scutellum (sc), coleoptile (col), and shoot apical meristem (sam), reaching to the coleoptilar stage, and the endosperm was large and filled normally (Figure 5A). But the *ppr101-1* embryo had undifferentiated embryo proper still attached to a suspensor, and its endosperm development was also hindered (Figure 5D). At 15 DAP, the wildtype embryo was well-developed and formed visible sc, col, coleorhizae (cor), sam, leaf primordia (lp) and root apical meristem (ram), reaching to the maturation stage. And the endosperm further developed, forming a plump starch filled endosperm tightly and wrapped by pericarp (Figures 5B,C). In *ppr101-1*, instead, the embryo remained at the transition stage with no obvious growth and differentiation, and the endosperm was small and underdeveloped (Figures 5E,F).

In *ppr231-1*, the arrest of seed development was not as severe as *ppr101-1*. At 9 DAP, the wildtype embryo reached to the coleoptilar stage (Figure 5G), whereas the mutant embryo development was delayed at the transition stage (Figure 5J). At 15 DAP, the wildtype embryo differentiated well with distinguishable sc, col, sam, lp, ram and cor, reaching to late embryogenesis stage (Figures 5H,I). In contrast, the mutant embryo was remained at the coleoptilar stage with indistinct sc and col (Figures 5K,L). Moreover, the endosperm development of *ppr231-1* was slower than that of the WT at 9 and 15 DAP. These results indicate that the mutation of two *PPR*s hampers seed development and results in embryo lethality.

### PPR101 and PPR231 Are Required for the Splicing of Mitochondrial Introns

**FIGURE 6:**
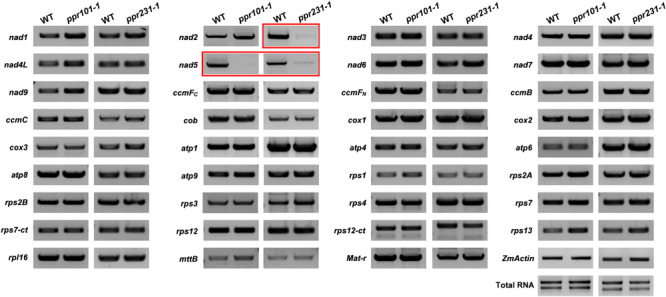
RT-PCR analysis of expression level of 35 mitochondrial transcripts in the WT and mutants. The level of *nad5* transcript is dramatically reduced and nearly undetectable in *ppr101-1*. And the level of *nad2* and *nad5* transcripts is decreased substantially in *ppr231-1*. The RNA was isolated from the same ear segregating for the wildtype and mutant kernels at 12 DAP. Expression was normalized with total RNA and *ZmActin.*

P-type PPR proteins have been reported to participate in organelle RNA metabolism such as intron splicing, RNA stabilization, and translation (Barkan and Small, 2014). To uncover the function of the two PPRs, we compared the expression level of mitochondrial transcripts of 35 protein coding genes between the WT and mutants. Specific primers used to amplify the transcripts are listed in Supplementary Table S1. RT-PCR analysis indicates that the level of *nad5* transcript is dramatically reduced and nearly undetectable in *ppr101-1*. And the *nad2* and *nad5* transcript levels are decreased substantially in *ppr231-1*. No significant differences were detected in the level of other mitochondrial transcripts between the WT and mutants (Figure 6).

**FIGURE 7:**
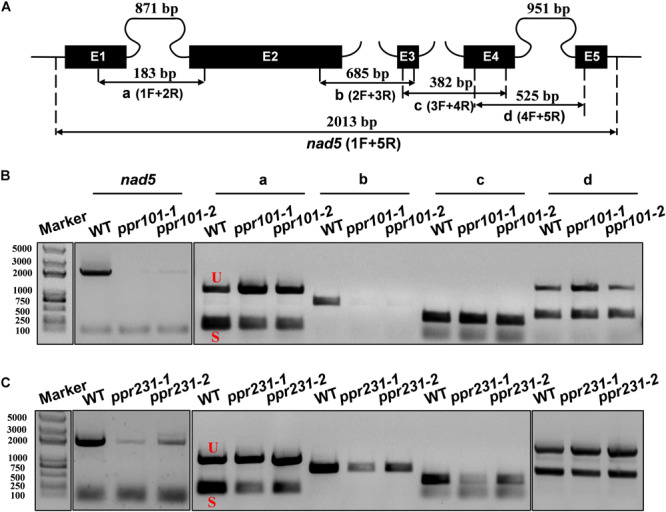
PPR101 is required for the splicing of *nad5* introns 1 and 2, and PPR231 for *nad5* introns 1, 2, 3. **(A)** Structure of the maize mitochondrial *nad5* gene. Exons are indicated with black boxes. The closed and opened lines stand for *cis*-spliced and *trans*-spliced introns. The expected amplification products using different exon flanking primers are indicated (a–d). E1-E5: exon1-exon5. **(B,C)** RT-PCR analysis of intron-splicing deficiency of *nad5* in the WT, *ppr101* and *ppr231* mutants. RNA was isolated from two alleles of mutant and wildtype kernels at 12 DAP. Exon–exon primers are used as indicated in panel **(A)**. S and U indicate the spliced and unspliced PCR products, respectively.

**FIGURE 8:**
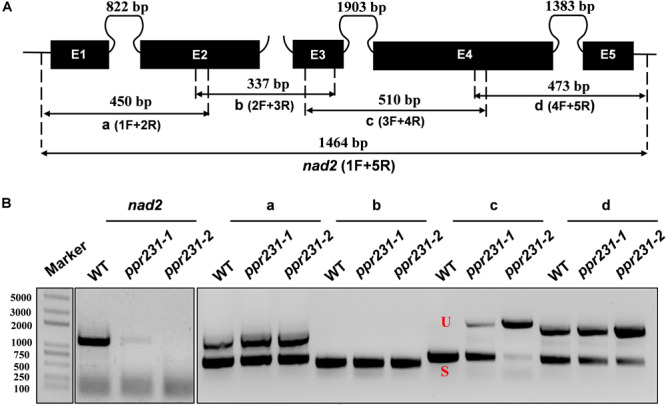
PPR231 is also essential to the splicing of *nad2* intron3. **(A)** Structure of the maize mitochondrial *nad2* gene. Exons are indicated with black boxes. The closed and opened lines stand for *cis*-spliced and *trans*-spliced introns. The expected amplification products using different exon flanking primers are indicated (a–d). E1-E5: exon1-exon5. **(B)** RT-PCR analysis of intron-splicing deficiency of *nad2* in the WT *and ppr231* mutants. RNA was isolated from two alleles of *ppr231* mutant and wildtype kernels at 12 DAP. Exon–exon primers are used as indicated in panel **(A)**. S and U indicate the spliced and unspliced PCR products, respectively.

Maize mitochondrial *nad5* has two *trans*-splicing introns (introns 2 and 3) and two *cis*-splicing introns (introns 1 and 4) (Figure 7A). *nad2* contains one *trans*-splicing intron (intron 2) and three *cis*-splicing introns (introns 1, 3 and 4), (Figure 8A; Brown et al., 2014). The decrease of *nad2* and *nad5* transcripts in mutants may be caused by intron splicing defects. To test this possibility, RT-PCR was performed with primers anchored on neighboring exons across each intron. The results show that in the *ppr101* mutants, the splicing of *nad5* intron 1 is reduced and the splicing of *nad5* intron 2 is nearly abolished, whereas the splicing of introns 3 and 4 is normal (Figure 7B). In the *ppr231* mutants, the splicing of *nad5* introns 1, 2, and 3 is decreased (Figure 7C). In addition, the *cis*-splicing of *nad2* intron 3 is also reduced with the accumulation of unspliced fragment (Figure 8B). The larger fragments were sequenced and confirmed the presence of unspliced introns. PPR proteins are proposed to bind RNA nucleotides via the one PPR motif recognizing one ribonucleotide mechanism where the sixth amino acid of the first PPR motif and the first amino acid of the next PPR motif specify one nucleotide (Barkan et al., 2012; Takenaka et al., 2013). Based on these codes and with the aid of a web program^4^, we predicted the putative binding sequences of PPR101 and PPR231 with the target intron sequences (Supplementary Figure S3). The positions of these putative binding sites in the intron were marked in relative position to the donor or receptor site. An attempt to map these sequences on the intron domains was given up due to the high viability in assigning intron domains and unavailable *trans*-intron breaking ends.

**FIGURE 9:**
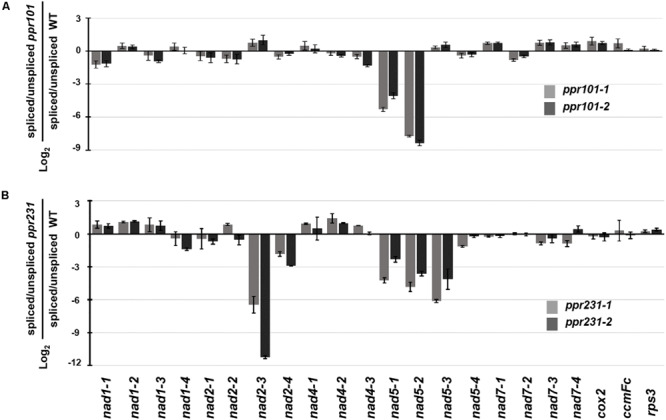
Splicing efficiency analysis in the *ppr101* and *ppr231* mutants. Quantitative RT-PCR analysis of all 22 group II introns in maize mitochondrial genes in the *ppr101* **(A)** and *ppr231* **(B)** mutants. Total RNA was isolated from two alleles of the *ppr101* and *ppr231* mutant and wildtype kernels at 12 DAP. Values is calculated by the log_2_ ratio of spliced to unspliced forms for each intron in two mutants compared with the WT. Values represent the mean and standard deviation of three biological replicates, ±SD.

To independently verify the splicing defects in the *ppr101* and *ppr231* mutants, we performed qRT-PCR to examine the splicing efficiency of all the 22 group II introns in maize mitochondrial genome (Figure 9). Spliced and unspliced transcripts were amplified specially with two sets of primers. The splicing efficiency was calculated by the ratio of spliced to unspliced products of each transcript in the mutants normalized to the same ratio in the WT. Results indicate that the splicing efficiency of *nad5* introns 1 and 2 is decreased in the *ppr101* mutants (Figure 9A), and the splicing efficiency of *nad5* introns 1, 2 and 3, and *nad2* intron 3 is reduced in the *ppr231* mutants (Figure 9B). No significant alteration in the splicing efficiency of other mitochondrial group II introns was detected. The RT-PCR and qRT-PCR results confirm that PPR101 is essential to the splicing of *nad5* introns 1 and 2, whereas PPR231 is critical to the splicing of *nad5* introns 1, 2, 3 and *nad2* intron 3.

### Loss of Function in PPR101 and PPR231 Impairs Mitochondrial Complex I Assembly and Activity

**FIGURE 10:**
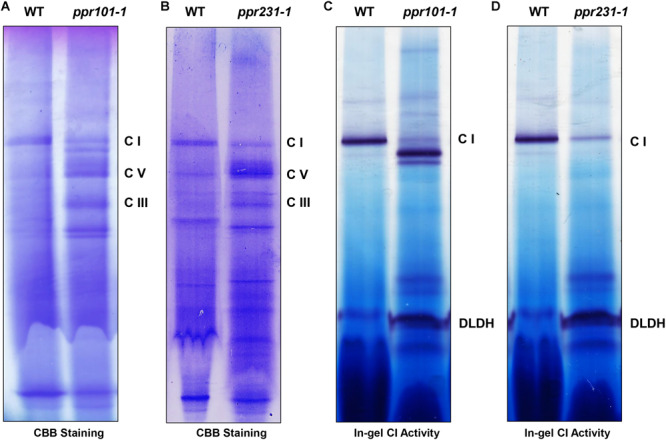
The assembly and activity of mitochondrial complex I are affected in the *ppr101-1* and *ppr231-1* mutants. **(A,B)** Blue native polyacrylamide gel electrophoresis (BN-PAGE) analysis of mitochondrial complexes assembly in *ppr101-1* **(A)** and *ppr231-1* **(B)**. Crude mitochondrial proteins were isolated and separated according to Sun et al. (2015). The BN gel was stained with Coomassie Brilliant Blue (CBB). C I, complex I; C III, complex III; C V, complex V. **(C,D)** NADH dehydrogenase activity assay of complex I in *ppr101-1* **(C)** and *ppr231-1* **(D)**. Dihydrolipoamide dehydrogenase (DLDH) was used as a loading control.

Complex I is composed of two arms, the “membrane arm” that embeds in the membrane and “peripheral arm” that protrudes into the matrix (Peters et al., 2013). Nad2 and Nad5 are key subunits of the membrane arm, hence the deficiency in *nad2* and *nad5* transcripts is predicted to affect mitochondrial complex I biogenesis. To assess the impact of *PPR101* and *PPR231* mutations on the complexes, we performed blue native polyacrylamide gel electrophoresis (BN-PAGE) and in-gel NADH dehydrogenase activity assays. Results showed that the WT and mutants exhibited a different pattern of complex proteins (Figure 10). Coomassie Brilliant Blue (CBB) staining showed that complex I was dramatically decreased while complex III and V were increased in two mutants compared with the WT (Figures 10A,B). In-gel NADH dehydrogenase activity assays indicated that the complex I activity disappeared at the holo-complex I position in *ppr101-1*, but appeared at positions of a smaller size, suggesting a possible accumulation of sub-complex I (Figure 10C). The *ppr231-1* mutant also showed a reduction in the activity of complex I but did not show the NADH dehydrogenase activity in the sub-complexes, suggesting that such sub-complex I cannot be assembled in *ppr231-1* (Figure 10D).

**FIGURE 11:**
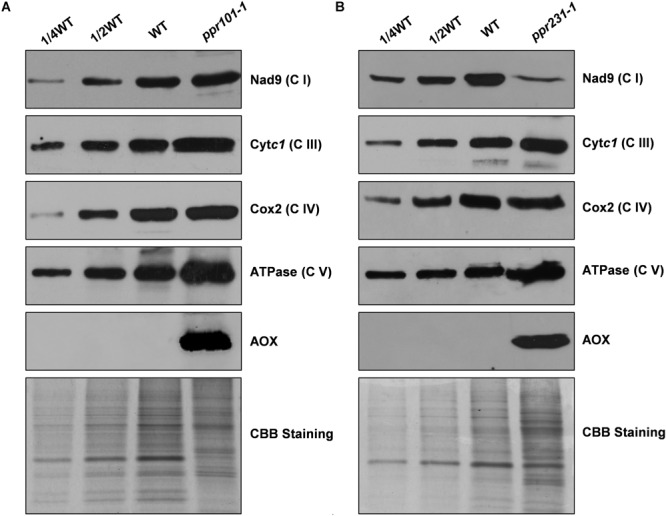
Accumulation of mitochondrial proteins in the *ppr101-1* and *ppr231-1* mutants. Western blotting analysis of mitochondrial proteins in the *ppr101-1* **(A)** and *ppr231-1* **(B)** with antibodies against Nad9, Cytc*1*, Cox2, ATPase, and AOX. Crude mitochondrial proteins of the WT and mutants were separated by SDS-PAGE and transferred to nitrocellulose membrane. CBB-stained gel was used as reference of loading quantity.

To examine the impact on protein abundance of mitochondrial complexes, western blotting assay was performed with specific antibodies against representative proteins of different mitochondrial complexes including Nad9 (complex I), Cyt*c*1 (complex III), Cox2 (complex IV), and α subunit of ATPase (complex V). The results showed that the Nad9 (Complex I) level was dramatically decreased in *ppr231-1* and but not in *ppr101-1*. A slight increase was detected in the level of Cyt*c*1 and Cox2, but substantial increase was occurred in the level of ATPase in both mutants (Figure 11). These results together with the BN-PAGE and in-gel assays indicate that the loss-of-function mutation of *PPR101* and *PPR231* reduces the activity and assembly of complex I, which may affect the expression of components in other mitochondrial complexes.

### Alternative Respiratory Pathway Is Activated in *ppr101-1* and *ppr231-1*

The block of complex I has been shown to activate the expression of alternative oxidase (AOX) in the alternative pathway (De Longevialle et al., 2007; Toda et al., 2012). We then analyzed the expression of AOX at the RNA and protein level. The maize genome contains three *AOX* genes, *AOX1* (AY059646.1), *AOX2* (AY059647.1), and *AOX3* (AY059648.1). RT-PCR and qRT-PCR results indicate that in two mutants, the expression level of *AOX2* transcript is upregulated by 6 to 16 times in comparison with the WT (Supplementary Figure S4). Consistently, the AOX protein as detected by specific antibody was induced dramatically in two mutants (Figure 11). These results provide further evidence that the mutation of either *PPR101* or *PPR231* impairs complex I biogenesis, leading to dysfunction of mitochondrial respiratory chain.

## Discussion

### PPR101 and PPR231 Function in Intron Splicing in Maize Mitochondria

Through a genetic and molecular study, we revealed the molecular function of PPR101 and PPR231. PPR101 is required for the splicing of *nad5* introns 1 and 2, and PPR231 for the splicing of *nad5* introns 1, 2, 3, and *nad2* intron 3 in mitochondria. This conclusion is supported by (1) All two proteins are localized in mitochondria; (2) two alleles of each mutant show identical defects in the splicing of the specific *nad5* and *nad2* introns; and (3) the deficiency in the splicing of *nad5* and *nad2* introns is corroborated by the dysfunction in the assembly and activity of mitochondrial complex I.

P-type PPR proteins have been implicated in RNA splicing, RNA stabilization and translation in plant organelle (Barkan and Small, 2014). We have not detected any other localization of these two proteins in compartments other than mitochondria although PPR proteins have been reported to localize in nucleus or dual-localize in chloroplasts and mitochondria. It is also unlikely that these two proteins have functions of RNA C-to-U editing although P-type PPR proteins also have been reported to function in RNA C-to-U editing (Doniwa et al., 2010; Leu et al., 2016). We sequenced all 35 gene transcripts in mitochondria and found no defect in C-to-U editing in two mutants.

In maize, several P-type PPR proteins have been reported to function in mitochondrial intron splicing. The loss of DEK2 leads to the decrease of *nad1* intron 1 splicing efficiency and causes small kernels (Qi et al., 2017). EMP8 participates in the *trans*-splicing of *nad1* intron 4 and *cis*-splicing of *nad4* intron 1 and *nad2* intron 1, and is responsible for mitochondrial complex I biogenesis (Sun et al., 2018). EMP16 is required for the *cis*-splicing of *nad2* intron 4, the mutation of which influences the complex I assembly and activates the expression of *AOX2*, resulting in an empty pericarp phenotype (Xiu et al., 2016). DEK37 was reported to be required for the *nad2* intron 1 splicing and seed development (Dai et al., 2018). The mutation of *PPR20* impairs the splicing of *nad2* intron 3, arresting the development of embryo and endosperm (Yang et al., 2019). EMP12 is implicated in the splicing of three *nad2* introns and indispensable to complex I biogenesis (Sun et al., 2019). The loss-of-function of DEK41 affects the splicing of *nad4* intron 3 and seed development (Zhu et al., 2019). EMP602 functioning on the splicing of *nad4* introns 1 and 3, is associated with the formation of mitochondria and assembly of complex I (Ren et al., 2019). These studies indicate that PPR proteins play an essential role in seed development. Hence, characterizing more PPRs by reverse genetics approach will uncover the function of PPR proteins and elucidate the molecular mechanism of seed development.

### PPR101 and PPR231 Are Essential to Complex I Biogenesis and Seed Development in Maize

Complex I is the major entry point for the respiratory chain and is located at the inner membrane of mitochondria. Nad2 and Nad5 are considered as antiporter-like subunits, similar to potential proton translocation sites, and are essential to the function of complex I (Efremov and Sazanov, 2011). Previous studies have reported about the splicing deficiency of *nad2* or *nad5* introns affecting complex I biogenesis. In Arabidopsis, the mutation of *mTERF15* which is required for *nad2* intron3 splicing reduced the activity and assembly of complex I (Hsu et al., 2014). The loss-of-function of RUG3 responsible for the splicing of *nad2* introns 2 and 3 impairs the complex I biogenesis (Kuhn et al., 2011). *Tang2* and *Otp439* are involved in *nad5* introns splicing. The mutation of two genes almost blocks the assembly and activity of complex I (Francs-Small et al., 2012). In maize, the *Emp10*, *Emp12*, *Emp16*, *PPR20* are required for *nad2* introns splicing. The mutants of these genes all generate impaired complex I biogenesis (Xiu et al., 2016; Cai et al., 2017; Sun et al., 2019; Yang et al., 2019). In our study, the defects in the splicing of *nad2* and *nad5* introns which resulted in the lack of Nad2 and Nad5, impede the assembly and activity of complex I in two mutants (Figure 10). Results suggest the significant role of PPR101 and PPR231 in *nad2* and *nad5* introns splicing and the complex I biogenesis.

As Complex I is essential to mitochondrial function, the defect of complex I will hazard the mitochondrial respiratory chain and cause detrimental consequence to seed development (Noctor et al., 2007). Therefore, the block of complex I biogenesis may has close correlation with seed development. Our results indicated that the assembly and activity of complex I reduced to different extent in *ppr101-1* and *ppr231-1* (Figure 10), thus resulting in different phenotypes of two mutants. NADH oxidase activity at the position expected for complex I was almost undetectable in *ppr101-1*, which generated the *empty pericarp* phenotype. In *ppr231-1*, complex I activity was reduced but still detectable which was not as severe as the *ppr101-1*, resulting in the *small kernel* phenotype. In previous studies, different degree of inhibition of complex I activity producing different maize kernel phenotypes also happened. In *emp8*, *emp10*, *emp11*, *emp12*, *emp16* and *emp602* mutants, the activity of complex I is almost abolished, which dramatically arrest the embryogenesis and endosperm development, thus generating *empty pericarp* maize kernels (Xiu et al., 2016; Cai et al., 2017; Ren et al., 2017, 2019; Zhang et al., 2017; Sun et al., 2018). However, the complex I activity in *smk1*, *dek2*, and *dek37* mutants is merely reduced, which alleviates the arrestment of embryo and endosperm development, producing a *small kernel* phenotype (Li et al., 2014; Qi et al., 2017; Dai et al., 2018). Therefore, results highlight the essential function of PPR101 and PPR231 in intron splicing, mitochondrial complex I biogenesis and seed development in maize.

### Characterization of the Two Mutants Sheds Lights to the Regulation of Complex I Subunit Accumulation

While all these two mutants are deficient of complex I, however, the impact on the accumulation of Nad9, a component of complex I, is different. Nad9 is accumulated to a wildtype level in *ppr101-1*, but drastically decreased in *ppr231-1* (Figure 11). Our data suggest that the decreased Nad9 accumulation in *ppr231-1* is not caused by either a decreased transcription or defect in the translation machinery. Because *nad9* is normally transcribed (Figure 6), and Cyt*c*1, Cox2 and ATPase which are encoded by the mitochondria are translated normally in *ppr231*-1 (Figure 11). The same phenomenon was also reported in previous study. In two *PPR* mutants, *mtl1* and *mtsf1*, which were defective in the *nad7* mRNA translation and *nad4* transcript stability, respectively, the function of complex I respiratory was impaired and the level of Nad9 translation was decreased about 2-fold in two mutants. However, compared with the WT, the level of *nad9* transcript was unchanged in *mtl1* and increased slightly in *mtsf1*. This suggests that the dysfunction of complex I may cause a negative feedback regulation on the level of Nad9 specifically (Planchard et al., 2018).

The reduced level of Nad9 is probably caused by protein degradation. The degradation of Nad9 in *ppr231-1* may imply a regulatory mechanism on the turnover of Nad9. Owing to defects in intron splicing, the *ppr101* mutant is deficient of Nad5, whereas the *ppr231* mutant is deficient of Nad5 and Nad2. This indicates that deficiency of both Nad2 and Nad5 or Nad2 alone can trigger the degradation of Nad9. Previous studies have indicated that the deficiency of Nad2 itself will dramatically reduce the accumulation of Nad9 in maize. The *emp16* mutant which is deficient in the splicing of *nad2* intron 4 cannot accumulate Nad9 (Xiu et al., 2016), the *emp12* mutant which is defective in the splicing of *nad2* introns 1, 2 and 4 cannot accumulate Nad9 either (Sun et al., 2019), and the *ppr20* mutant which is defective in the splicing of *nad2* intron 3 also abolish the accumulation of Nad9 (Yang et al., 2019). Instead, defects in components of other mitochondrial complexes as in maize *emp9*, *emp18* and *smk4* mutants do not affect Nad9 accumulation (Yang et al., 2017; Li et al., 2019; Wang et al., 2019). Also defects in other subunits of complex I, as in Arabidopsis *nmat4* and *ppr19* mutants, the *nad1* intron splicing deficiency blocks the complex I activity dramatically, do not reduce Nad9 abundance (Cohen et al., 2014; Lee et al., 2017). These results suggest a regulatory mechanism exists in plants where a deficiency of Nad2 triggers the degradation of Nad9 in maize mitochondria.

Evidence suggests that this regulatory mechanism may be related to the assembly process of complex I in plants. A recent study has shown that the assembly of complex I follows a modular pathway in Arabidopsis (Ligas et al., 2019). In this pathway, Nad2 assembles into the main P_P_ module, Nad5 assembles into the P_D_ module, and Nad9 assembles into the matrix arm via the Q module. Then the P_P_ module assembles with the matrix arm to form complex I^∗^, and sequentially assembles with the P_D_ module to form the complete complex I (Ligas et al., 2019). Although the assembly of complex I in maize is not yet known, the present data suggest that defects in the P_D_ module as in *ppr101* mutant do not trigger the degradation of Nad9 in the Q module, but defects in the P_P_ module do which is observed in *ppr231* mutant. It is possible that when the Q module cannot assemble into the P_P_ module, Q module subunits are degraded, which include Nad7, Nad9, and other proteins. Following this reasoning, it would be interesting to check the accumulation of all the Q module matrix arm subunits in two mutants. Unfortunately, we do not have the antibodies.

This conclusion is also supported by our results on complex I assembly. In *ppr101* mutant, the formation of sub-complex I was detected, but not in *ppr231* mutant (Figure 10). The sub-complex I may be the assembled P_P_ module with the matrix arm but without the P_D_ module according to the pathway in Arabidopsis (Ligas et al., 2019).

### Splicing of Single Intron Requires Multiple Splicing Factors in Mitochondria

PPR101 and PPR231 are required for multiple introns splicing, and meanwhile, they have functional overlap in the splicing of specific introns. Specifically, they are all required for the splicing of *nad5* introns 1 and 2. As previously reported, in Arabidopsis, a CRM protein, mCSF1 is participated in the splicing of *nad5* introns 1, 2, 3, and possibly 4 (Zmudjak et al., 2013); OTP439 and TANG2, two PPR proteins are involved in the splicing of *nad5* introns 2 and 3, respectively (Colas et al., 2014). In maize, ZmSMK9 is required for the splicing of *nad5* introns 1 and 4 (Pan et al., 2019). These results imply that the splicing of a single intron requires multiple splicing factors.

It seems that these splicing factors can be divided into two categories. The first class, like CRM, RNA helicases and intron maturases can be taken as general splicing factors which function in multiple group II introns splicing. They may constitute the core of a spliceosome, and therefore are essential to mitochondrial introns removal. The second class of splicing factors include PPR proteins, like OTP439, TANG2, SMK9, PPR101, and PPR231. They are likely to perform as assistant factors in recruiting other mitochondrial splicing factors and/or sustaining the stability and improving the activity of this spliceosome when splicing on specific introns.

Previous studies have pointed out that PPRs have the capability to form complexes, and they may cooperate to form a specific spliceosome in mitochondrial introns splicing, such as PPR10 (Yin et al., 2013), PNM1 (Hammani et al., 2011), and GRP23 (Ding et al., 2006). PNM1 is reported to be involved in a 120 kDa complex (Hammani et al., 2011; Senkler et al., 2017). GRP23 is identified in a 160 kDa complex including PMH2 and nMAT2 in mitochondria (Senkler et al., 2017; Zmudjak et al., 2017). As PPR101 and PPR231 overlap in the splicing of a specific *nad5* intron, it is reasonable to conjecture that they have interaction. However, yeast two-hybrid assay revealed no direct interaction between them (Supplementary Figure S5). These phenomena also happened in previous studies. EMP12 and EMP16 are implicated in *cis-*splicing of *nad2* intron 4 but fail to interact physically (Sun et al., 2019). Similarly, PPR20 and Zm_mTERF15 are all specifically involved in *nad2* intron 3 splicing. But there is no interaction between them (Yang et al., 2019).

The molecular mechanism of PPR proteins in splicing is still ambiguous, but possible reasons with respect to this are speculated as follows: (1) They function independently in a complex without physical interaction directly. (2) They probably have interaction, but like the nuclear spliceosome, they constitute a highly dynamic complex, which acts transiently or unstably on multiple introns splicing. (3) They facilitate intron splicing not by protein-protein interaction. As PPRs embrace different RNA-binding domain, they can bind intron sequences independently at different sites, help introns folding correctly and maintain the configuration of intron sequences in a catalytically active state without interaction.

## Data Availability Statement

The datasets generated for this study are available on request to the corresponding author.

## Author Contributions

HY and B-CT designed the research. HY performed most of the experiments. ZX and XL participated in subcellular localization experiments. LW performed the western blotting assay. S-KC and FS participated in the BN gel experiment. HY, ZX, and B-CT analyzed the data. HY and B-CT wrote the manuscript.

## Conflict of Interest

The authors declare that the research was conducted in the absence of any commercial or financial relationships that could be construed as a potential conflict of interest.

## Footnotes

1 http://www.thermofisher.com

2 https://www.qiagen.com/cn/

3 http://www.bio-rad.com

4 http://yinlab.hzau.edu.cn/pprcode
