# Supplementary material for: Two Pentatricopeptide Repeat Proteins Are Required for the Splicing of nad5 Introns in Maize
Source: Front Plant Sci. 2020 Jun 3;11:732. doi: 10.3389/fpls.2020.00732 (PMC7284535; doi:10.3389/fpls.2020.00732)
Supplement: Supplementary file 1 [file Data_Sheet_1.PDF]

Supplementary Material

Figure S1:

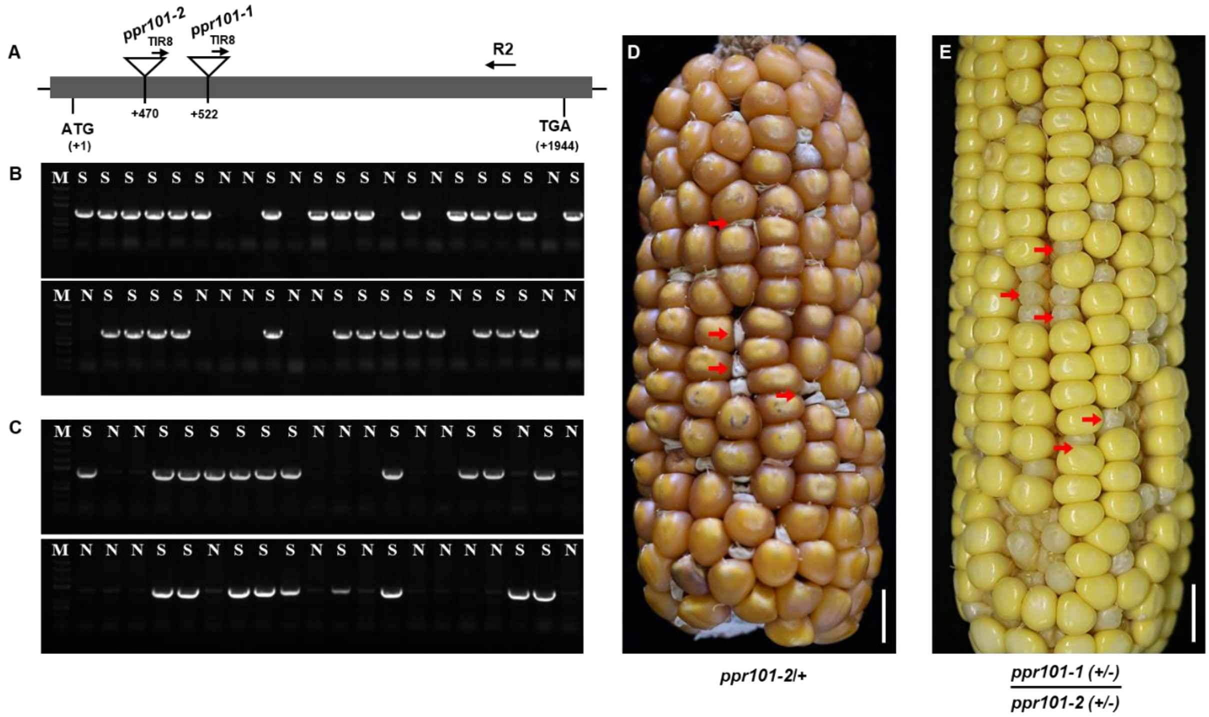

Figure S1. Linkage analysis of *empty pericarp* phenotype and *Mu* insertions in *PPR101*

(A) *PPR101* gene structure and position of *Mu* insertions. Primers indicated are Mu-TIR8 and *PPR101* specific primer, PPR101-R2 for linkage analysis.

(B, C) Linkage analysis of *PPR101-1* (B) and *PPR101-2* (C) population with primers TIR8 and PPR101-R2. M, marker; N, nonsegregation (WT); S, segregation (heterozygote).

(D) Self-pollinations of *ppr101-2* heterozygote segregate *emp* kernels as indicated by red arrows. Scale bar = 1 cm.

(E) Crosses between *ppr101-1*/+ and *ppr101-2*/+ produce ears segregating *emp* kernels at a 1:3 ratio (*emp*: WT). Red arrows indicate *emp* mutant kernels. Scale bar = 1 cm.

Figure S2:

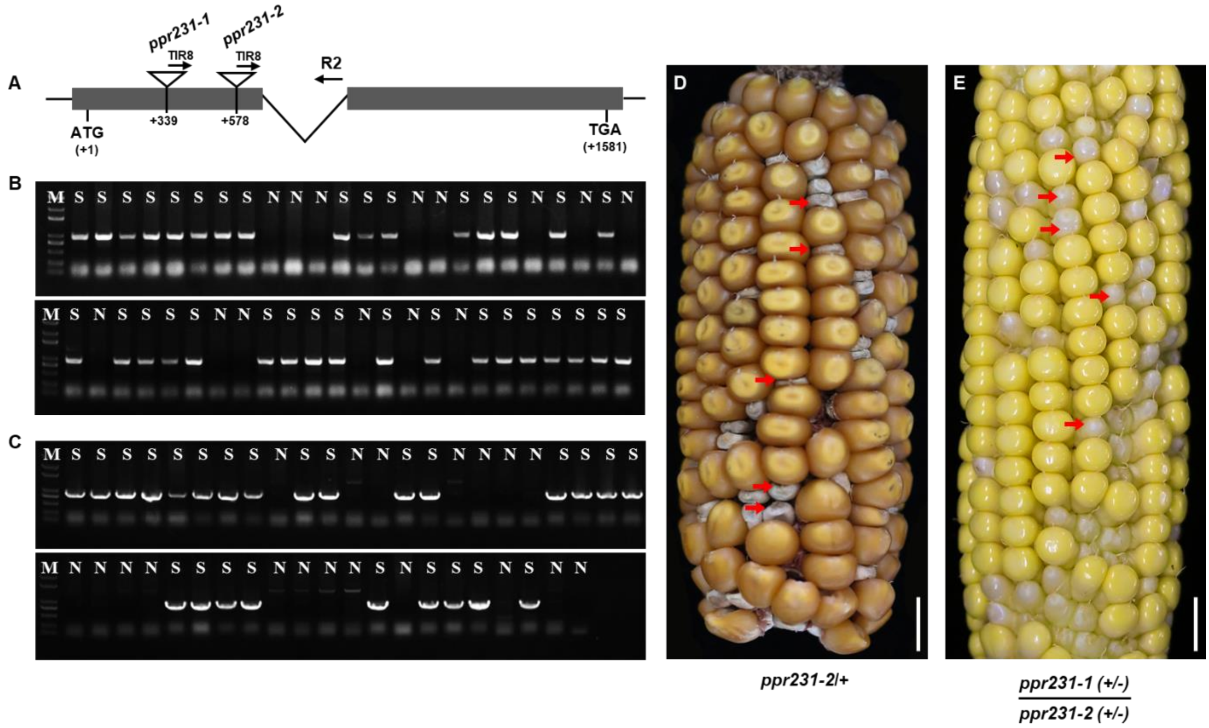

Figure S2. Linkage analysis of phenotype and *Mu* insertions in *PPR231*

(A) *PPR231* gene structure and position of *Mu* insertions. The primers are Mu-TIR8 and *PPR231* specific primer, PPR231-R2.

(B, C) Linkage analysis of *PPR231-1* (B) and *PPR231-2* (C) population with primers TIR8 and PPR231-R2. M, marker; N, nonsegregation (WT); S, segregation (heterozygote).

(D) Self-pollinations of *ppr231-2* heterozygote segregated *seed-defective* kernels as indicated by red arrows. Scale bar = 1 cm.

(E) Crosses between *ppr231-1*/+ and *ppr231-2*/+ produce ears segregating *smk* kernels at a 1:3 ratio (*smk*: WT). Red arrows indicate mutant kernels. Scale bar = 1 cm.

Figure S3:

A

PPR motif	P	P	P	P	P	P	P	P	P	P	P	P	P	P	P	P	Position
PPR 101	E	V	N	N	A	N	T	T	N	S	T	N	N	N	S	T	6
	T	P	N	D	V	N	N	S	N	N	N	N	S	N	N	N	1’
nad5 intron1	c	a	c	c	a	c	a	u	c	a	u	u	g	c	a	a	+813
nad5 intron2	a	a	g	u	g	a	a	c	a	a	g	u	g	u	a	a	+249
RNA base	N	N	c=u	u/c	N	c=u	a	a/g	c=u	a	a	c=u	c/u	c=u	a	a	

B

PPR motif	P	P	P	P	P	P	P	P	P	Position
PPR 231	D	A	S	N	S	T	N	N	T	6
	P	N	N	D	T	S	A	D	Q	1’
nad2 intron 3	u	a	a	u	a	a	a	g	a	-466
nad5 intron 1	g	a	a	u	a	a	c	u	c	+390
nad5 intron 2	c	a	a	u	a	a	g	u	g	+1097
nad5 intron 3	a	a	a	u	a	a	g	g	c	-899
RNA base	N	a/u	a	u/c	a	a/g	N	u/c	N	

Figure S3. Predicted binding sites of PPR101 and PPR231 in introns.

The combinatorial amino acid codes for RNA recognition at positions 6 and 1’ in PPR motifs of PPR101 **(A)** and PPR231 **(B)** are predicted by the website (http://yinlab.hzau.edu.cn/pprcode). The binding sequence of *nad5* introns 1, 2 and 3, and *nad2* intron 3 is determined by the frequencies of co-occurrence of amino acids at positions 6, 1’ and nucleotides of introns (Barkan et al., 2012; Takenaka et al., 2013). The stronger matches are shaded in green, the moderate matches are shaded in yellow, and the mismatches are in red. “P” stands for classical PPR motifs. The numbers mean the relative positions of these putative binding sites to the donor site (+1) or receptor site (-1) in the intron.

Figure S4:

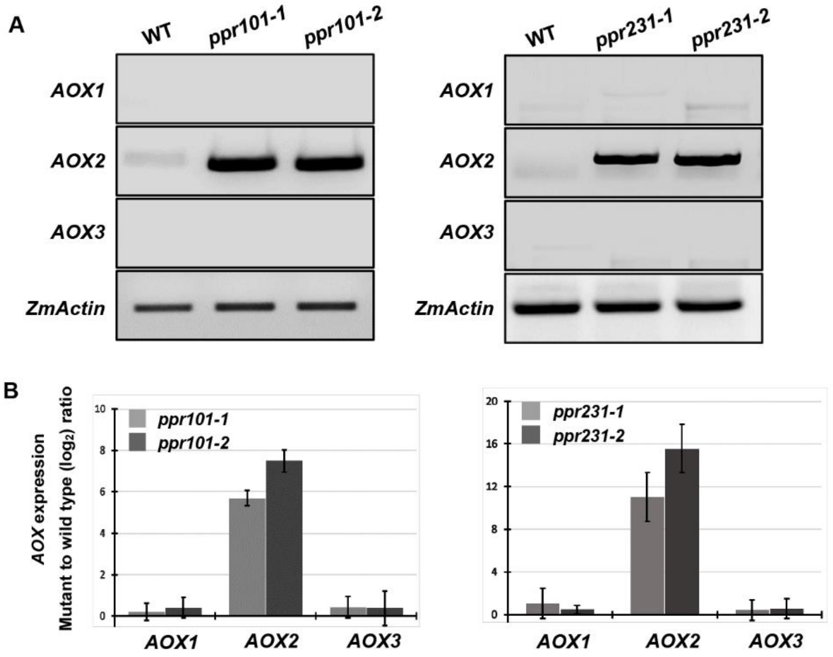

Figure S4. Expression of *AOX2* gene dramatically increased in the *ppr101* and *ppr231* mutants

(A) RT-PCR analysis of *AOX1*, *AOX2* and *AOX3* expression level in the WT, *ppr101*and *ppr231* mutants. Total RNA was extracted from kernels at 12 DAP after pericarp removal. *ZmActin* was used as normalization.

(B) Quantitative RT-PCR analysis of *AOX1*, *AOX2* and *AOX3* expression in the WT, *ppr101* and *ppr231* mutants at 12 DAP. *ZmActin* was used as the internal control. Values are means of three biological replicates, ±SD. AOX, alternative oxidase.

Figure S5:

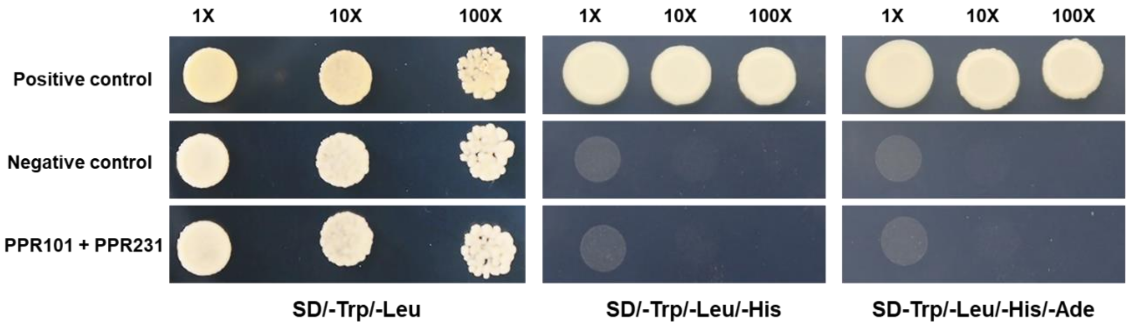

Figure S5. PPR101 and PPR231 have no interaction with each other in yeast two-hybrid assay.

The interaction between PPR101 and PPR231 was identified by yeast two-hybrid. Mature PPR101 and PPR231 without signal peptide were co-transformed and yeast cells were sprayed on dropout medium of DDO (SD/-Leu/-Trp), TDO (SD/-Leu/-Trp/-His) and QDO (SD/-Leu/-Trp/-His/-Ade). Blank vectors of AD (pGADT7) and BD (pGBKT7) and positive controls were co-transformed according to the manual of Clontech.

Table S1. Primers used in this study.

Primer name	Primer sequence (5' to 3')	Use for
TIR8a	CGCCTCCATTTCGTCGAATCCCCTS	PCR, genotype *ppr101* mutant
TIR8b	CGCCTCCATTTCGTCGAATCCSCTT	PCR, genotype *ppr101* mutant
TIR8c	SGCCTCCATTTCGTCGAATCCCKT	PCR, genotype *ppr101* mutant
TIR8d	CGCCTCCATTTCGTCGAATCACCTC	PCR, genotype *ppr101* mutant
PPR101-F1	CAACCATTTGTGGCATCAG	PCR, genotype *ppr101* mutant<br>RT-PCR, analysis of *PPR101*gene expression
PPR101-R1	TGCAGAACGCATCGACTA	RT-PCR, analysis of *PPR101*gene expression
PPR101-R2	ATCGCACCTAACATCCAAGC	PCR, genotype *ppr101* mutant
PPR101-RT-F1	AGGTGTTCTCAGTTGTCAGCG	qRT-PCR, expression pattern of *PPR101*
PPR101-RT-R1	CCTCCACATGTCTAGTGCGTC	qRT-PCR, expression pattern of *PPR101*
PPR101-CF	AATAGCATCATCTTCTCGACATTC	Cloning full length *PPR101*
PPR101-CR	AACTTTGCCTTCATTGGGAT	Cloning full length *PPR101*
PPR101-LF	CACCATGTGTTCGATAAAATGCTGCTTCCTC	Construct PPR101$^{\mathbf{N200}}$: GFP vector
PPR101-LR	CGTCACGTCGTCCGGGGGACA	Construct PPR101$^{\mathbf{N200}}$: GFP vector
PPR101-Y2H-F	GGAATTCCATATGACCGGGGACGCGGCGAGAGCC	Construct PPR101 yeast two hybrid vector
PPR101-Y2H-R	CGGGATCCTCAGGTCCCTTCTGATAAGTT	Construct PPR101 yeast two hybrid vector
PPR231-F1	AATACTGTGCCTGTCGAAGCC	PCR, genotype *ppr231* mutant<br>RT-PCR, analysis of *PPR231* gene expression
PPR231-R1	TGTCACTCCGATTCACCTTGC	RT-PCR, analysis of *PPR231* gene expression
PPR231-R2	AAGATTAGAGAAATAGCAGAGCA	PCR, genotype *ppr231* mutant
PPR231-RT-F1	GCACCCTGATTACCGATTTGAT	qRT-PCR, expression pattern of *PPR231*
PPR231-RT-R1	TTTCACCATCTTCCAATCTCCC	qRT-PCR, expression pattern of *PPR231*
PPR231-CF	CATCGGCTAAATCGGACTC	Cloning full length *PPR231*
PPR231-CR	CCAGTTCCTCCACCACTATCTT	Cloning full length *PPR231*
PPR231-LF	CACCATGCCGCCGCCTCTCCC	Construct PPR231$^{\mathbf{N205}}$: GFP vector
PPR231-LR	CTTGGGGTACTTGTACTTGCA	Construct PPR231$^{\mathbf{N205}}$: GFP vector
PPR231-Y2H-F	GGAATTCCATATGCTCGCGCGCCTCCTCCTCGCG	Construct PPR231 yeast two hybrid vector
PPR231-Y2H-R	CGGGATCCTCACCTCTTGTAAGGCTTCAT	Construct PPR231 yeast two hybrid vector
nad1-F	GGCCCGATCATGAGTGAATA	RT-PCR, gene expression analysis
nad1-R	GCCCCCTTCAGAAGAAACTT	RT-PCR, gene expression analysis
nad2-F	GACGGAGGAGAGGAAATGAA	RT-PCR, gene expression analysis
nad2-R	GCAGTCCACCCTTTCTTTGA	RT-PCR, gene expression analysis
nad3-F	CTTTCCTATGTCCTTCCCCC	RT-PCR, gene expression analysis
nad3-R	GAGGAGAGCGAGAGAACGAA	RT-PCR, gene expression analysis
nad4-F	CAGTCACCCGGAGAAGATTT	RT-PCR, gene expression analysis
nad4-R	TAATTTGGCGCCTGATTGAC	RT-PCR, gene expression analysis
nad4L-F	CTGACATTCCATGTTTCCGA	RT-PCR, gene expression analysis
nad4L-R	GAAGAGAACGAAAGGAGAACAGA	RT-PCR, gene expression analysis
nad5-F	CGCTCGAACATTGTCTGATT	RT-PCR, gene expression analysis

nad5-R	GTCCTGGCAAGCTCCTACAG	RT-PCR, gene expression analysis
nad6-F	TGGAAAAACCAAACCCACAT	RT-PCR, gene expression analysis
nad6-R	CAAGTTCCCTTGGCGTAGTC	RT-PCR, gene expression analysis
nad7-F	GTTTTGGCTCGCAATAAAGC	RT-PCR, gene expression analysis
nad7-R	CAGGTGGGACAAGCTCTAGG	RT-PCR, gene expression analysis
nad9-F	AGCAAGAAGCGGAACAAAAA	RT-PCR, gene expression analysis
nad9-R	TATTGATTTGTCCCCTCCCC	RT-PCR, gene expression analysis
rps1-F	AAGGTGGGCTTCGGATTATT	RT-PCR, gene expression analysis
rps1-R	TCTTCAGTTTTACGCTTACGCT	RT-PCR, gene expression analysis
rps2A-F	CAGGAAAGATATTTGCCCCA	RT-PCR, gene expression analysis
rps2A-R	CCTGTATCTCCGGAAACGAA	RT-PCR, gene expression analysis
rps2B-F	TCCATGGACCCACGTAAAAT	RT-PCR, gene expression analysis
rps2B-R	GGCCCCTCTCTGATAAGGAA	RT-PCR, gene expression analysis
rps3-F	GCAGAAAGGGGCAAAAGTAA	RT-PCR, gene expression analysis
rps3-R	TCGCGACCCCTACTACATCT	RT-PCR, gene expression analysis
rps4-F	AGAGTTGGGTTCGATTCCCT	RT-PCR, gene expression analysis
rps4-R	AGCGACTAGGCCGATCTTTT	RT-PCR, gene expression analysis
rps7-F	TTCGTTGGAAAAACCTACGC	RT-PCR, gene expression analysis
rps7-R	ATGAGGAAGGCCGATTTTCT	RT-PCR, gene expression analysis
rps7-ct-F	TTGAACCTCTTTCACGCTCA	RT-PCR, gene expression analysis
rps7-ct-R	TTCCGATCGAGATGTATGGA	RT-PCR, gene expression analysis
rps12-F	CTAGCTGCTTCCATATCGCC	RT-PCR, gene expression analysis
rps12-R	CGGATCGGGAGTAACCACTA	RT-PCR, gene expression analysis
rps12-ct-F	TGTACGGTTCTGTAGAGGGACA	RT-PCR, gene expression analysis
rps12-ct-R	TCCGTTTTCTTTTTATAAGGGC	RT-PCR, gene expression analysis
rps13-F	TCATGATGATTAAGGGAAGAGTGA	RT-PCR, gene expression analysis
rps13-R	TTGAATTGAACAGTGTGATTGAT	RT-PCR, gene expression analysis
rpl16-F	GGTTTTTCCCCACTAACCAA	RT-PCR, gene expression analysis
rpl16-R	GGGTGCGGAAATAGCTAGAA	RT-PCR, gene expression analysis
atp1-F	CGTTGCTGGTGAAGAAGCAT	RT-PCR, gene expression analysis
atp1-R	AAAAGCGGATTTATCCATCG	RT-PCR, gene expression analysis
atp4-F	AGCCACGTGCTCTAATCCTC	RT-PCR, gene expression analysis
atp4-R	TCCCTTTCTCTTGGAGCAGA	RT-PCR, gene expression analysis
atp6-F	CCAAGTCTCTTTTGGGAGCA	RT-PCR, gene expression analysis
atp6-R	GGCTCCTCGTTTTTATGCAA	RT-PCR, gene expression analysis
atp8-F	GGCAAGGATCCTCAGTCCTA	RT-PCR, gene expression analysis
atp8-R	GAGGGTTGGTTTGATTGGAA	RT-PCR, gene expression analysis
atp9-F	AGGGGCCTCGTCATCTCTAT	RT-PCR, gene expression analysis
atp9-R	TAGTTGCGAAGGAAAAGCGT	RT-PCR, gene expression analysis
ccmB-F	AGCCGTCGAAGTGAATGAAT	RT-PCR, gene expression analysis
ccmB-R	AACGGCTTTTCCATGACTTG	RT-PCR, gene expression analysis
ccmC-F	ACTTGCAAGGCAAGGAAAAA	RT-PCR, gene expression analysis
ccmC-R	CCATGGATGCTTTAGCGAGT	RT-PCR, gene expression analysis

ccmFC-F	GAGAAGCTCAAATCGAACGG	RT-PCR, gene expression analysis
ccmFC-R	CGCAGCCACTATTTTGACTC	RT-PCR, gene expression analysis
ccmFN-F	TGAAGATTGTAAGGCGTTTCC	RT-PCR, gene expression analysis
ccmFN-R	GGATCATCCTGTGGTTACCG	RT-PCR, gene expression analysis
cob-F	ATCAAGGCAAGGGGGTAAAT	RT-PCR, gene expression analysis
cob-R	GGTGTGATCAGTCTCATCCG	RT-PCR, gene expression analysis
cox1-F	GGCCCCTCTCTGATAAGGTT	RT-PCR, gene expression analysis
cox1-R	GTTAAGGCAAAGCCCAAACA	RT-PCR, gene expression analysis
cox2-F	GTCCTACTTCTGGTGCTGCC	RT-PCR, gene expression analysis
cox2-R	GAGAATTGCATTTCCGCTTC	RT-PCR, gene expression analysis
cox3-F	TCAATCCACTTATTCGTTCCC	RT-PCR, gene expression analysis
cox3-R	GTTTACATACAACCGGGGCA	RT-PCR, gene expression analysis
Mat-r-F	AACGCCTGTTCGCTAAAATC	RT-PCR, gene expression analysis
Mat-r-R	AGGCTTTGCTCCCCTTTTT	RT-PCR, gene expression analysis
mttB(orfX)-F	TTGGTTTAGAATTGCTCGGG	RT-PCR, gene expression analysis
mttB(orfX)-R	AGGGGGAACCCTACCGAC	RT-PCR, gene expression analysis
ZmActin-F	ATGGTCAAGGCCGGTTTCG	ZmActin abundance
ZmActin-R	TCAGGATGCCTCTCTTGGCC	ZmActin abundance
nad1-1F	GCAACGTCGAAAGGGTCCTG,	Test *nad1* intron 1 splicing efficiency by RT-PCR
nad1-2R	TGAGCTGCAGATCGTAATGC	
nad1-2F	TCGAAATATGCCTTTCTAGGAG	Test *nad1* intron 2 splicing efficiency by RT-PCR
nad1-3R	ATTCAGCTTCCGCTTCTGG	
nad1-3F	GTCATGGCGCAAAAGCAGATATGG	Test *nad1* intron 3 splicing efficiency by RT-PCR
nad1-4R	AGAGCAGACCCCATTGAAGA	
nad1-4F	TCTTCAATGGGGTCTGCTCT	Test *nad1* intron 4 splicing efficiency by RT-PCR
nad1-5R	AGGGAGCCATCGAAAGGTGA	
nad2-1F	GACGGAGGAGAGGAAATGAA	Test *nad2* intron 1 splicing efficiency by RT-PCR
nad2-2R	GCCGGGATCATTAAGAGCATAC	
nad2-2F	CTCGCAGTATGCTCTTAATGATCC	Test *nad2* intron 2 splicing efficiency by RT-PCR
nad2-3R	GGAACTGCAGTAATCTTGAATAGGG	
nad2-3F	TCTACTGGAGCTACCCACTTCGA	Test *nad2* intron 3 splicing efficiency by RT-PCR
nad2-4R	GGTTTGCCGTAATGCTGGA	
nad2-4F	TTCCAGCATTACGGCAAACC	Test *nad2* intron 4 splicing efficiency by RT-PCR
nad2-5R	GCAGTCCACCCTTTCTTTGA	
nad4-1F	GGTCCTATTCTCTGTCCCGTGC	Test *nad4* intron 1 splicing efficiency by RT-PCR
nad4-2R	GTAAATCGGTGGTTCCTGTTTGG	
nad4-2F	TCATTATAGGGGTATGGGGTTCG	Test *nad4* intron 2 splicing efficiency by RT-PCR
nad4-3R	CTAGTGCCGGGTAAACTCATATTG	
nad4-3F	TAGTCCGAACATACCGGGAATTG	Test *nad4* intron 3 splicing efficiency by RT-PCR
nad4-4R	CTTACGGATGTATGCATGCAGTC	
nad5-1F	CGCTCGAACATTGTCTGATT	Test *nad5* intron 1 splicing efficiency by RT-PCR
nad5-2R	AGCAGATACTGGAGTGGGAC	
nad5-2F	GTCACTGCTGGCGTTTTC	Test *nad5* intron 2 splicing efficiency

nad5-3R	TACCTAAACCAATCATCATATC	by RT-PCR
nad5-3F	GATATGATGATTGGTTTAGGTA	Test *nad5* intron 3 splicing efficiency by RT-PCR
nad5-4R	GCCAATCGTCGGAATGTG	
nad5-4F	TTGCCGAATCCGAGTTTG	Test *nad5* intron 4 splicing efficiency by RT-PCR
nad5-5R	GTCCTGGCAAGCTCCTACAG	
nad7-1F	TAATTTGGCGCCTGATTGAC	Test *nad7* intron 1 splicing efficiency by RT-PCR
nad7-2R	CTCGATTAATTTCTCAGTCCCTC	
nad7-2F	GAGGGACTGAGAAATTAATCGAG	Test *nad7* intron 2 splicing efficiency by RT-PCR
nad7-3R	CTCGACATAAGCCAAGAGGC	
nad7-3F	GCCTCTTGGCTTATGTCGAG	Test *nad7* intron 3 splicing efficiency by RT-PCR
nad7-4R	CCGAACACTTTGTCGCATCT	
nad7-4F	AGATGCGACAAAGTGTTCGG	Test *nad7* intron 4 splicing efficiency by RT-PCR
nad7-5R	GTTTTGGCTCGCAATAAAGC	
cox2-1F	GTCCTACTTCTGGTGCTGCC	Test *cox2* intron splicing efficiency by RT-PCR
cox2-2R	GAGAATTGCATTTCCGCTTC	
rps3-1F	GCAGAAAGGGGCAAAAGTAA	Test *rps3* intron splicing efficiency by RT-PCR
rps3-1R	CAGAGCGGGACTTCTTTGGTA	
ccmFC-1F	CGATAGGTCAGCGAAGCGTG	Test *ccmFC* intron splicing efficiency by RT-PCR
ccmFC-1R	AGACCTCGCAAACAACAACGT	
nad1-int1F	TGTCAGATCCGAACATAGGG	Test spliced *nad1* exon1-2
nad1-int1R	TGCAGATCGTAATGCTCCTAGA	
nad1-exonF1	TATGTTAAGTCTGGTCGCTTGGG	Test unspliced *nad1* exon1-int1
nad1-intronR1	TATATCATAGGCGACCGAACGG	
nad1-int2F	TGCAGCTCAAATGGTCTCTT	Test spliced *nad1* exon2-3
nad1-int2R	AATACGGGGAACAAGGGAAT	
nad1-exonF2	TCGAAATATGCCTTTCTAGGAG	Test unspliced *nad1* exon2-int2
nad1-intronR2	AAAACTCAAAACGAGCCTTGCG	
nad1-int3F	ATTCCCTTGTTCCCCGTATT	Test spliced *nad1* exon3-4
nad1-int3R	AAAAGAGCAGACCCCATTGA	
nad1-exonF3	GTCATGGCGCAAAAGCAGATATGG	Test unspliced *nad1* exon3-int3
nad1-intronR3	GAATGAGTCCCGAGACATTGGC	
nad1-int4F	TCCCCGTATTGGTTATGTTCC	Test spliced *nad1* exon4-5
nad1-int4R	GATCATATTGGCATACTCTCCC	
nad1-exonF4	GGGAGAGTATGCCAATATGATCTTA	Test unspliced *nad1* exon4-int4
nad1-intronR4	GAGTCAAAGGGTCACCACTACTGAG	
nad2-int1F	AGTAATGTGGGTTGGCTTGG	Test spliced *nad2* exon1-2
nad2-int1R	GAAATGGTACCAGCCGTACTT	
nad2-exonF1	GCGGTTTCCCCAGAGATCTTTC	Test unspliced *nad2* exon1-int1
nad2-intronR1	TACGATTAGCCAGCCTTGCGGC	
nad2-int2F	TCGCAGCATCAAAAAGAAAG	Test spliced *nad2* exon2-3
nad2-int2R	GATCGAAGTGGGTAGCTCCA	
nad2-exonF2	TGATCTTAGGTGCATTTCCCTCTG	Test unspliced *nad2* exon2-int2
nad2-intronR2	ATCGGTAGTAGTCCGGTCGCAC	

nad2-int3F	ACCGGATACGAAATCACTGG	Test spliced *nad2* exon3-4
nad2-int3R	GCGCAATAGAAAGGAATGCT	
nad2-exonF3	TCTACTGGAGCTACCCACTTCGA	Test unspliced *nad2* exon3-int3
nad2-intronR3	AGCGGTACCACCCATCCTACC	
nad2-int4F	GGTTGTGGGGCTTACTTCCT	Test spliced *nad2* exon4-5
nad2-int4R	CGACTTGTCACGATCCATTG	
nad2-exonF4	TTCCAGCATTACGGCAAACC	Test unspliced *nad2* exon4-int4
nad2-intronR4	TACTCATGGCAACCTTCCGGC	
nad4-int1F	GGTGGTTCCTGTTTGGAGAA	Test spliced *nad4* exon1-2
nad4-int1R	AGCGTGCCAATCCCTATGT	
nad4-exonF1	ATGATCGCCGTGTCCTGCATGC	Test unspliced *nad4* exon1-int1
nad4-intronR1	AAGCTTCGCGGGGACCTTGAC	
nad4-int2F	GAAGATCATTGCCTACTCCTCA	Test spliced *nad4* exon2-3
nad4-int2R	AGGGCTGAAGAAACCAGTCC	
nad4-exonF2	CAGTAGCCCATATGAATTTGGTG	Test unspliced *nad4* exon2-int2
nad4-intronR2	CGCTAAGGGGTTTTGTTTTAGG	
nad4-int3F	GTGAACACCCATCCGAACA	Test spliced *nad4* exon3-4
nad4-int3R	GGCGTATTCCCTTTGGCTAT	
nad4-exonF3	TACCCGGCACTAGCAGCTTTATC	Test unspliced *nad4* exon3-int3
nad4-intronR3	CCCATCGCAAGCACCTACAATG	
nad5-int1F	CCATGGATCTCATCGGAAAT	Test spliced *nad5* exon1-2
nad5-int1R	CACATAAATCGAGGGCTATGC	
nad5-exonF1	ATCTCAGAATAGCTCCATGGATCTC	Test unspliced *nad5* exon1-int1
nad5-intronR1	CGGGAGTTGTTACGTCCAGTATG	
nad5-int2F	TTTGCTTTCTGGTTGGGAAG	Test spliced *nad5* exon2-3
nad5-int2R	TCATATCTTTGGCCAAGTATCCTAC	
nad5-exonF2	AGAGCTCGCTTACACAAAGTATACC	Test unspliced *nad5* exon2-int2
nad5-intronR2	TACTTACTTATGGGCTAACAGGTCAC	
nad5-int3F	GATTGGTTTAGGTACAATTTTTGG	Test spliced *nad5* exon3-4
nad5-int3R	TTTGAAAGGCTCGTTGGAAT	
nad5-exonF3	GATATGATGATTGGTTTAGGT	Test unspliced *nad5* exon3-int3
nad5-intronR3	TTTTCCTCAGTTGCAGGGTTTG	
nad5-int4F	CGTACACATTCCGACGATTG	Test spliced *nad5* exon4-5
nad5-int4R	CCCACATACGAGAAAAGGTCA	
nad5-exonF4	AAGGGTGCTATTGAGATATTGGG	Test unspliced *nad5* exon4-int4
nad5-intronR4	CTTTCCTCGGGTTCGTAGAGTC	
nad7-int1F	CGGGCAAATCAAGAATTTCA	Test spliced *nad7* exon1-2
nad7-int1R	CTCGATTAATTTCTCAGTCCCTCT	
nad7-intronF1	GGATTTGCGAATGAATGCTG	Test unspliced *nad7* int1-exon2
nad7-exonR2	CTCGATTAATTTCTCAGTCCCTC	
nad7-int2F	TCAAGCTTTACCTTATTTTGATCG	Test spliced *nad7* exon2-3
nad7-int2R	TGATGCTCCCACATCCATAG	
nad7-intronF2	GTTGTTCGTTCCGTCGTTGA	Test unspliced *nad7* int2-exon3

nad7-exonR3	CTCGACATAAGCCAAGAGGC	
nad7-int3F	GATTGGGGATTCAGTGGTGT	Test spliced *nad7* exon3-4
nad7-int3R	CGAACACTTTGTCGCATCTC	
nad7-exonF3	GCCTCTTGGCTTATGTCGAGATA	Test unspliced *nad7* exon3-int3
nad7-intronR3	ATGGGAACTTCCCCCATATTGC	
nad7-int4F	CCATCACGATCTCGAATGAA	Test spliced *nad7* exon4-5
nad7-int4R	TAGGTGCTTCAACTGCGGTA	
nad7-exonF4	AGATGCGACAAAGTGTTCGGAT	Test unspliced *nad7* exon4-int4
nad7-intronR4	TTTACTCCTAACCCCACGACGG	
ccmFc-F2	TTATTTCGTTTCGTTCCCGTTC	Test spliced *ccmFc* exon1-2
ccmFc-R2	TGTTCAAACATGAACCTTTCGC	
ccmFc-exonF1	CGACTGTTGATGGCTGTTGGTC	Test unspliced *ccmFc* exon1-int1
ccmFc-intronR1	GTCAACTGAGCATCTCAGCGGC	
cox2-int1F	CTCAATGGACGGGGTATTAG	Test spliced *cox2* exon1-2
cox2-int1R	CACAAAGAGCGATTGTGAGG	
cox2-exonF1	AGCTATTGGACATCAATGGTATCG	Test unspliced *cox2* exon1-int1
cox2-intronR1	CGGGGTATAGGTCTAACCACCTC	
rps3-sense	CAGATCCAAGTCGGTTCAGTGA	Test spliced *rps3* exon1-2
rps3-antisense2	AGTCTCGTAGGTGGACGTATCG	
rps3-exonF1	TTTCGGTAAGACTTGATCTGAATCG	Test unspliced *rps3* exon1-int1
rps3-intronR1	CTTTCACGACATGCTCTGGTCC	
